# Diverting stomas reduce reoperation rates for anastomotic leak but not overall reoperation rates within 30 days after anterior rectal resection: a national cohort study

**DOI:** 10.1007/s00384-022-04205-8

**Published:** 2022-06-24

**Authors:** Elisabeth Myrseth, Linn Såve Nymo, Petter Fosse Gjessing, Stig Norderval

**Affiliations:** 1 Department of Gastrointestinal Surgery, University Hospital of North Norway, 9019 Tromsø, Norway; 2 Institute of Clinical Medicine, Faculty of Health Science, UiT, The Arctic University of Norway, 9019 Tromsø, Norway

**Keywords:** Stoma, Anterior resection, Anastomotic leak, Rectal cancer

## Abstract

**Purpose:**

A diverting stoma is commonly formed to reduce the rate of anastomotic leak following anterior resection with anastomosis, although some studies question this strategy. The aim of this study was to assess the leak rates and overall complication burden after anterior resection with and without a diverting stoma.

**Methods:**

A 5-year national cohort with prospectively registered data of patients who underwent elective anterior resection for rectal cancer located < 15 cm from the anal verge. Data were retrieved from the Norwegian Registry for Gastrointestinal Surgery and the Norwegian Colorectal Cancer Registry. Primary end point was relaparotomy or relaparoscopy for anastomotic leak within 30 days from index surgery. Secondary endpoints were postoperative complications including reoperation for any cause.

**Results:**

Some 1018 patients were included of whom 567 had a diverting stoma and 451 had not. Rate of reoperation for anastomotic leak was 13 out of 567 (2.3%) for patients with diverting stoma and 35 out of 451 (7.8%) (p > 0.001) for patients without. In multivariable analyses not having a diverting stoma (aOR 3.77, c.i 1.97–7.24, p < 0.001) was associated with increased risk for anastomotic leak. However, there were no differences in overall reoperation rates following anterior resection with or without diverting stoma (9.3% vs 10.9%, p = 0.423), and overall complication rates were similar. Reoperation was associated with increased mortality irrespective of the main intraoperative finding.

**Conclusion:**

Diverting stoma formation after anterior resection is protective against reoperation for anastomotic leak but does not affect overall rates of reoperation or complications within 30 days.

## Introduction

Anastomotic leak following anterior resection for rectal cancer is a major complication, leading to increased morbidity, prolonged hospital stay, additional interventions and in some cases death [1, 2]. Even if the anastomosis can be rescued for some patients, leaks are associated with inferior functional results with lifelong implications for the patient [3–5]. The reported leak rate after anterior resections varies between 6.5% and 13.6% [6–9], and one reason for this variation might be differences in definition and grading of severity of anastomotic leaks. Rabhari et al. [10] proposed in 2010 criteria for standardized definitions. The authors defined three categories of leaks where grade A leaks do not require any intervention, grade B leaks require active intervention but without relaparotomy, and grade C leaks require relaparotomy or relaparoscopy.

In order to prevent anastomotic leak, formation of a temporary diverting stoma is common following resections with low anastomoses, and two recent meta-analyses have shown lower leak rates in patients receiving diverting stomas [11, 12]. Norwegian guidelines [13] recommend diverting ileostomy in case of anastomosis < 7 cm from anal verge based on results from the Norwegian Colorectal Cancer Registry [14]. Consideration of a diverting stoma following low anterior resection (LAR) is also recommended by the Association of Coloproctology of Great Britain and Ireland [15], but the recommendation does not define a specific group of patients for which stomas should be considered. Nevertheless, stoma-related morbidity and complications represent a significant problem [16–18], and this should warrant selection of patients at risk for anastomotic leak before diverting stoma is considered. Furthermore, there is an ongoing debate whether diverting stomas only mask possible anastomotic leak and further delay the diagnosis. A Swedish multicenter trial showed that only 60% of the leaks after LAR were diagnosed during the initial hospital stay [19], and a Dutch multicenter study showed that half of the late diagnosed leaks never heal [20].

The aim of this study was to assess the anastomotic leak rates and overall complication rates after anterior resection with and without a diverting stoma in a national cohort from the Norwegian Registry for Gastrointestinal Surgery (NoRGast) [21] linked with data from the Norwegian Colorectal Cancer Registry [22]. Primary endpoint was reoperation for anastomotic leak within 30 days after anterior resection with and without diverting stomas. The dataset did not allow for exploration of anastomotic leak or stoma rate later than 30 days after index surgery. Secondary endpoints were overall complication rates including reoperation of any cause.

## Methods

### Study population

**Fig. 1:**
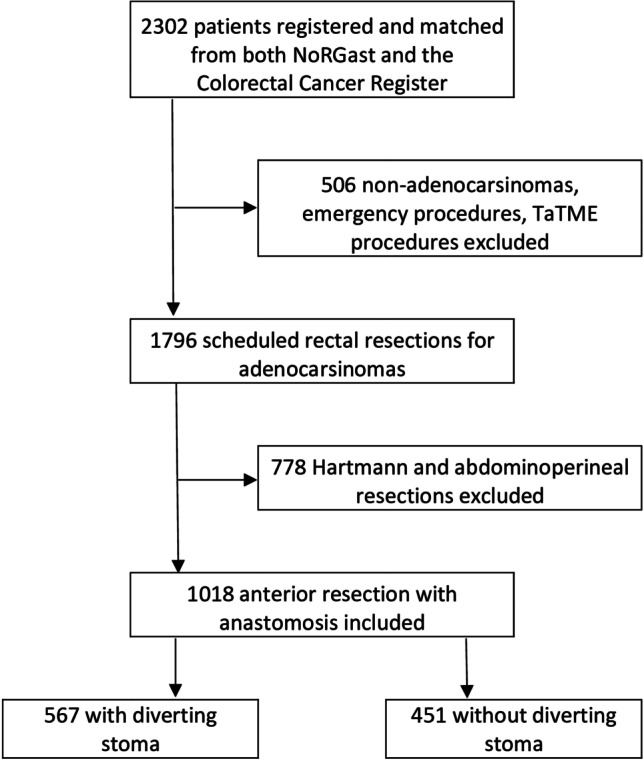
Flowchart

Patients who underwent elective major resection for rectal cancer from January 1^st^ 2014 to December 31^st^ 2018 were identified via NoRGast based on procedure codes according to NCSP (NOMESCO Classification Of Surgical Procedures) [23] for rectal resections, and diagnosis code C20 for cancer according to the International Classification of Diseases version 10 (ICD-10) [24]. Tumors other than adenocarcinomas as well as endoscopic and TaTME procedures were excluded (Fig. 1). NoRGast is a national quality registry established in 2014 and records complications within 30 days after surgery. All Norwegian hospitals performing cancer resections are obliged to report data to NoRGast, and a detailed presentation of the registry has previously been published [21]. Data from NoRGast were linked via the patient’s individual social security numbers to the Norwegian Colorectal Cancer Registry [22] for information on preoperative work-up, neoadjuvant treatment and final histopathological results.

### Data quality

The national coverage rate in NoRGast has increased during the study period from 20% in 2014 to 75% in 2018 [25]. The low national coverage rate in 2014 was due to a limited number of participating hospitals the first year, although the coverage rates among the participating hospitals were high. The Norwegian Colorectal Cancer Registry has a coverage rate higher than 90% [26]. Variable completeness varies, with almost 100% completeness in NoRGast compared to 70% for some variables in the Norwegian Colorectal Cancer Registry. However, as both registries overlap on several core variables, data linking resulted in an overall high degree of variable completeness in the studied dataset. The correctness and reliability of variables in NoRGast is high much due to the digital reporting system, in which certain limitations and warnings for unusual combinations exist. The records are manually checked by local registrars 30 days postoperatively to increase validity. The manuscript was drafted in accordance to the STROBE guidelines for observational studies [27].

### Anastomotic leak definition

According to NoRGast, anastomotic leak was defined as a leak that required relaparotomy or relaparoscopy (grade C leak) [10] within 30 days after the index operation. The registry holds no data on less severe leaks (grade A or grade B leaks).

### Categorization of variables

There was no variable available in the register of whether a total mesorectal excision (TME) or a partial mesorectal excision (PME) had been performed. This is however closely related to tumor level. Hence tumor level was used as a proxy for TME and PME, respectively. Tumor level was measured preoperatively with a rigid proctoscope, and categorized into TME (tumor ≤ 12 cm from anal verge) and PME (tumor > 12 cm from anal verge). Age was categorized into three groups (low < 65 years, mid 65–80 years and high > 80 years). ASA-scores were grouped into low ASA-scores (scores 1–2), and high ASA-scores (scores 3–4). WHO ECOG-scores were dichotomized into low ECOG-score (0–1) and high ECOG-score (2–4). Severe pulmonary disease was defined as having FEV1 < 50 per cent or a vital capacity < 60 per cent of predicted values. Severe cardiac disease was defined as NYHA classification 3–4 or severe arrhythmia requiring mechanical support. Complications were registered according to the Accordion grading system [28], and major complications were defined as Accordion grade 3 or higher. The NoRGast registry categorized finding at reoperation as anastomotic leak, bleeding, deep infection without proof of leak, wound dehiscence and miscellaneous. Weight was classified by body mass index (BMI), and patients were grouped into 4 BMI-classes [29]; [< 18.5] [18.5–25] [25–30] [> 30]. Data were analyzed with SPSS version 26, (IBM, Armonk, New York, USA).

### Statistical analyses

For univariable analyses Pearson’s Chi-square test was used for categorical data, and two-sided T-test or Mann–Whitney U test for continuous data. Confidence interval (c.i.) or interquartile range (IQR) was calculated when appropriate. Univariable binary logistic regression was used to calculate unadjusted odds ratios (OR). To address and minimize the effects of possible bias resulting from differences in baseline characteristics between patient groups, a stepwise backwards multivariable logistic regression model with adjusted odds ratios (aOR) was used to further analyze the relations between different predictors and outcomes. Variables significant in univariable analyses at a level of p < 0.2 were included in multivariable analysis, and final significance level after multivariable analysis was set to p < 0.05. Relevant variables were tested for significant two-way interactions, and if interactions were found, they were further accounted for in the analyses. Little’s test [30] of whether data were missing completely at random was performed with all variables included for analyses in the test. The test had a Chi-square of 19.44, degrees of freedom = 13 and a non-significant p = 0.110 indicating that missing values were missing completely at random. This allowed patients with missing data in variables included for subgroup analyses to be excluded from these analyses.

The study was approved by The Regional Committee for Medical and Health Research Ethics (approval number 2018/2274) and by the Data Protection Officer at the University Hospital of North Norway.

## Results

### Patients

A total of 2302 patients were recorded in NoRGast with an NCSP procedural code for rectal resection during the study time frame. After excluding non-adenocarcinomas, TaTME, endoscopic and emergency procedures, a total of 1796 patients were identified, of whom 1018 patients underwent anterior resection with primary anastomosis. Some 742 of these 1018 operations were laparoscopic procedures including 191 robotic assisted procedures, and 276 were open access procedures (Fig. 1). Baseline characteristics for the included patients are presented in Table 1.

**Table 1:** Baseline characteristics, patients operated with anterior resection

Characteristics			Diverting stoma		
		*Total*	With	Without	P-value
Gender (F/M)		*398/620*	208/359	190/261	0.077
Age	< 65	*469*	284 (50.1%)	185 (41.0%)	< 0.001
	65–80	*477*	257 (45.3%)	220 (48.8%)	
	> 80	*72*	26 (4.6%)	46 (10.2%)	
BMI	< 18.5	*22*	10 (1.8%)	12 (2.7%)	0.385
	18.5–25	*393*	230 (41.8%)	163 (37.1%)	
	25–30	*405*	221 (40.2%)	184 (41.9%)	
	> 30	*169*	89 (16.2%)	80 (18.2%)	
ASA	1,2	*754*	422 (74.4%)	332 (73.8%)	0.814
	3,4	*263*	145 (25.6%)	118 (26.2%)	
ECOG	0,1	*958*	530 (94.6%)	428 (96.0%)	0.329
	2,3,4	*48*	30 (5.4%)	18 (4.0%)	
Pulmonary disease		*45*	23 (4.1%)	22 (4.9%)	0.526
Heart disease		*58*	32 (5.6%)	26 (5.8%)	0.934
Diabetes		*87*	54 (9.5%)	33 (7.3)	0.211
Access					
	Open	*276*	183 (66.3%)	93 (33.7%)	< 0.001
	Lap	*742*	384 (51.8%)	358 (48.2%)	
Tumor level					
	0–11,9 cm	*493*	364 (73.8%)	129 (26.2%)	< 0.001
	12,0–15,0 cm	*319*	94 (29.5%)	225 (70.5%)	
Radiochemotherapy		*239*	208 (87.0%)	31 (13.0%)	< 0.001

### Anastomotic leak rates

The overall leak rate was 48 out of 1018 (4.7%) with stratified rates for patients with and without a diverting stoma of 13 out of 567 (2.3%) and 35 out of 451 (7.8%) (p < 0.001), respectively. Leak rate was significantly lower with diverting stomas regardless of tumor level, and tumor level was not a significant predictor for anastomotic leak in univariable regression analyses. In multivariable regression analyses, absence of diverting stoma was associated with an increased risk of reoperation for anastomotic leak with an aOR of 3.77 (c.i. 1.97–7.24, p < 0.001) compared to anterior resection with a diverting stoma (Table 3).

### Complication rates

**Fig. 2:**
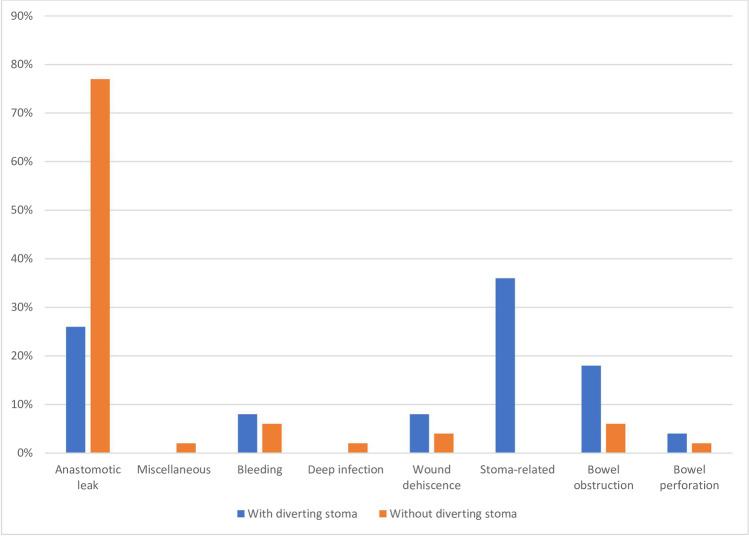
Main finding (%) at reoperation after anterior resection, with and without diverting stoma

The overall reoperation rate was 102 out of 1018 (10.0%). There was no difference in reoperation rates between the groups with and without diverting stomas (Table 2), but the findings at reoperation differed. For patients without a diverting stoma, the main finding at reoperation was anastomotic leak in 35 out of 47 (74.5%) patients, while anastomotic leak was the main finding at reoperation in 13 out of 51 (25.5%) patients with a diverting stoma (Fig. 2). Male gender (aOR 1.85) and severe pulmonary disease (aOR 3.44) were associated with increased risk of reoperation for any reason (Table 3). In NoRGast, patients with a diverting stoma were coded with main finding “miscellaneous” at reoperation in 58.8% of the cases in contrast to 12.8% of reoperations in patients without stoma. As a part of a registry quality review the electronical medical records for all patients coded with “miscellaneous” as main finding at reoperation were investigated and recategorized into more granular main findings. The review revealed that patients with a diverting stoma was reoperated due to stoma-related problems in 30.0% of the cases. Furthermore, bowel obstruction was the reason for reoperation in 18.0% of the patients with a diverting stoma compared to 6.4% in patients without diverting stomas (Table 2; Fig. 2).

**Table 2:** Results after anterior resection with or without diverting stoma

Results		Diverting stoma		
	Total	With	Without	P-value
Anastomotic leaks				
Open (276)	*16/276 (5.8%)*	4/183 (2.2%)	12/93 (12.9%)	< 0.001
Laparoscopy (742)	*32/742 (4.3%)*	9/384 (3.1%)	23/358 (6.4%)	0.006
Tumor level				
0–11,9 cm*	*1/11 (9.1%)*	12/364 (3.3%)	10/129 (7.8%)	0.035
12,0–15,0 cm*	*16/394 (4.1%)*	0/94 (0%)	17/22 (7.6%)	0.006
Reoperations	*102/1018 (10.0%)*	53/567 (9.3%)	49/451 (10.9%)	0.423
Finding at reoperation**				
Anastomotic leak	*49/97 (50.5%)*	13/50 (26.0%)	36/47 (76.6%)	< 0.001
Miscellaneous	*1/97 (1.0%)*	0 (0%)	1/47 (2.1%)	
Bleeding	*7/97 (7.2%)*	4/50 (8.0%)	3/47 (6.4%)	
Deep infection	*1/97 (1.0%)*	0/50 (0.0%)	1/47 (2.1%)	
Wound dehiscence	*6/97 (6.2%)*	4/50 (8.0%)	2/47 (4.3%)	
Bowel obstruction	*12/97 (12.4%)*	9/50 (18.0%)	3/47 (6.4%)	
Bowel perforation	*2/97 (2.1%)*	2/50 (4.0%)	1/47 (2.1%)	
Stoma-related	*18/97 (18.6%)*	15/50 (30.0%)	-	
Length of stay, median (IQR)	*6 (4–9)*	7 (5–10)	5 (4–8)	< 0.001
Major complications	*146/1018 (14.3%)*	87 (15.3%)	59 (13.1%)	0.306
90-day mortality	*14/1018 (1.4%)*	5 (0.9%)	9 (2.0%)	0.130
30-day mortality	*7/1018 (0.7%)*	4 (0.7%)	3 (0.7%)	0.938
Single-organ-failure	*25/1018 (2.5%)*	13 (2.3%)	12 (2.7%)	0.706
Multi-organ-failure	*6/1018 (0.6%)*	4 (0.7%)	2 (0.4%)	0.587

* Missing values = 206; ** Missing values = 2

**Table 3:** Results from multivariable regression analyses*

Outcome measure	Significant variables			Multivariable analyses	
	Variable		*Rate (%)*	aOR (95%CI)	p-value
Anastomotic leak	Gender				
		Female	*11/389 (2.8)*	Ref	0.012
		Male	*37/620 (6.0)*	2.43 (1.22–4.85)	
	Diverting stoma				
		Yes	*13/567 (2.3)*	Ref	< 0.001
		No	*35/451 (7.8)*	3.77 (1.97–7.24)	
Reoperation	Gender				
		Female	*27/398 (6.8)*	Ref	0.009
		Male	*75/620 (12.1)*	1.85 (1.17–2.94)	
	Severe pulmonary disease				
		Yes	*90/973 (9.2)*	Ref	< 0.001
		No	*12/45 (26.7)*	3.44 (1.71–6.94)	
30-day mortality	Age group				
		< 65	*1/469 (0.2)*	Ref	0.013
		65–80	*3/477 (0.6)*	2.13 (0.20–22.32)	
		> 80	*3/72 (4.2)*	19.99 (1.84–217.18)	
	Severe pulmonary disease				
		Yes	*4/973 (0.4)*	Ref	0.013
		No	*3/45 (6.7)*	8.41 (1.56–45.24)	
	Reoperation				
		Yes	*4/102 (3.9)*	12.42 (2.74–56.31)	0.004
		No	*3/916 (0.3)*	Ref	

^*^Variables included in univariable analyses: Age group, gender, WHO ECOG-score, ASA classification, severe pulmonary disease, severe cardiac disease, diabetes, weight class (BMI), operative access (open/laparoscopy), tumor level (TME/PME), preoperative radio(chemo)therapy, diverting stoma, anastomotic leak (not for analyses on anastomotic leak) and reoperation (not for analyses on reoperation)

The overall major complication rates, 30-day and 90-day mortality rates and rates of single-organ and multi-organ failure did not differ between the two groups (Table 2). Median LOS was 7 days in the group with diverting stoma compared to 5 days in the group without diverting stoma (p < 0.001).

There were no major differences in mortality or morbidity between patients reoperated for anastomotic leaks and patients reoperated for other reasons (Table 4), but LOS was longer following anastomotic leak (Table 4). In multivariable regression analyses, increasing age (65–80 years aOR 2.13 and > 80 years aOR 19.99), severe pulmonary disease (aOR 8.41) as well as reoperation (aOR 11.36) were associated with increased 30-day mortality risk (Table 3).

**Table 4:** Postoperative complications following reoperation for anastomotic leak and reoperation for other reason

	Anastomotic leak	Reoperation for other reasons	p-value
Length of hospital stay	Median 20 (IQR 14–27)	Median 17 (IQR 13–21)	0.039
90-day mortality	3/49 (6.1%)	2/48 (4.2%)	0.663
30-day mortality	2/49 (4.4%)	2/48 (4.2%)	0.983
Single-organ failure	6/49 (12.6%)	9/48 (18.8%)	0.376
Multi-organ failure	0/49 (0.0%)	3/48 (6.3%)	0.075

## Discussion

In the present study, reoperation for anastomotic leak within 30 days after anterior resection was significantly less frequent in patients with a diverting stoma. However, stoma diversion did not affect the overall reoperation rate, mortality or morbidity. This has to the authors knowledge not been shown in previous studies. Reoperation was associated with increased mortality irrespective of intraoperative finding, and the total burden of morbidity and mortality within 30 days were similar for patients with and without a diverting stoma.

The current evidence of the benefits of diverting stomas following anterior resection is unclear, and studies report diverging results. A recent meta-analysis showed lower anastomotic leak rates and reoperation rates with diverting stomas compared to no stomas [31], but the diagnostic criteria of leak and time to diagnosis varied in the included studies. A Swedish registry study of 1442 patients who underwent anterior resection showed that late presenting leaks were associated with diverting stomas, and that stoma formation did not alter the overall leak rate [32]. As many as 50% of the leaks were diagnosed after discharge, and about half of these patients needed relaparotomy. A Dutch multicenter study showed that half of the late diagnosed leaks never heal [20]. Several studies suggest that diverting stomas do not have any protective effect on late diagnosed leaks, and reoperation rate and permanent stoma rate seems to be high also after late diagnosed leaks [20, 32–34]. The functional results following anastomotic leak are inferior [35], but it is not known whether the severity of dysfunction differs after early and late discovered leaks. A Japanese study on 1903 patients who underwent LAR showed that formation of a diverting stoma did not protect against late diagnosed anastomotic leaks, and that permanent stoma rate was higher among patients with late diagnosed leaks compared to those with early diagnosed leaks [33].

Although diverting stomas apparently have a protective effect against early diagnosed leaks, several studies highlight the less favorable consequences of stoma formation [16–18]. A temporary stoma will in most cases lead to longer hospital stay and require a second operation and hospital stay for stoma closure. Additionally, patients may experience stoma leak, parastomal hernias, skin problems, dehydration, kidney failure and electrolyte deficiency which may require additional hospital visits.

In the present cohort diverting stomas did not lower morbidity, mortality or reoperation rates within the first 30 postoperative days. Reoperation for bleeding, deep infection and wound dehiscence was performed to the same extent regardless of whether the patient had received a diverting stoma or not. The patients who received a diverting stoma were also reoperated more frequently due to bowel obstruction, and 30% of the reoperations were directly stoma-related. In support of this notion, formation of diverting stomas has been shown to increase short-term complications including stoma related reoperations after anterior resection [16]. Furthermore, some studies report delayed stoma reversal, and that creation of a diverting stoma might increase risk of permanent stoma on long term [36, 37]

The results of the present study emphasize the question whether patients undergoing anterior resection derive any benefit from formation of a diverting stoma and if so, how to select these patients. As low tumor level did not represent a significant risk factor for anastomotic leak, the recommendation of diverting stoma formation for anastomosis level < 7 cm from anal verge can be challenged. To explore this issue further a long-term study on outcomes after anterior resection with and without diverting stomas is warranted, assessing both early and late diagnosed anastomotic leaks, long-term overall complication rates, permanent stoma rates and total length of hospital stay. A Norwegian multicenter trial, the Norwegian Stoma Trial, exploring some of these issues is planned for commencement in 2022 [38]. Furthermore, the ongoing Dutch IMARI [39] multicenter trial will explore the one-year anastomotic integrity rate before and after the introduction of a multi-interventional program aiming to reduce anastomotic leak rate. In this study, the impact of diverting stomas will also be accounted for.

This study has some limitations. NoRGast is a newly established register with low coverage rates during the first years of inclusion. As already described, causality between stoma related problems and indication for reoperation cannot be established due to the nature of the study. The present study is an observational registry study and it is possible that there are variables not registered that could have a confounding effect, and that there are factors not registered and hence accounted for that could lead to selection bias. Nevertheless, our findings add to the question whether the benefits of a diverting stoma following anterior resection is outweighed by the overall complication rate.
